# Non-invasive prediction of implantation window in controlled hyperstimulation cycles: Can the time from the menstrual day at embryo transfer to expected menstrual cycle give a clue?

**DOI:** 10.4274/tjod.34651

**Published:** 2016-09-15

**Authors:** İlhan Şanverdi, Enis Özkaya, Tayfun Kutlu, Taylan Şenol, Munip Akalın, Eda Sayar Akalın, Yavuz Şahin, Ateş Karateke

**Affiliations:** 1 Zeynep Kamil Women and Children’s Health Training and Research Hospital, Clinic of Obstetrics and Gynecology, İstanbul, Turkey

**Keywords:** Day of embryo transfer, artificial reproductive techniques, implantation

## Abstract

**Objective:**

The aim of this study was to assess whether the time from the menstrual day at embryo transfer to expected menstrual cycle (TETEMC) is associated with the implantation in women with regular cycles or not.

**Materials and Methods:**

Forty women with successful implantation and forty women without implantation with regular cycles were randomly selected from prospectively collected database of assisted reproductive technology clinic of Zeynep Kamil Women And Children’s Health Training and Research Hospital. TETEMC was calculated for each case to assess relationship with the successful implantation.

**Results:**

Comparison of groups revealed significant differences with regard to TETEMC and the menstrual period (p<0.05).

In ROC analyses both the TETEMC (AUC=0.824, p<0.001) and the menstrual period (AUC=0.797, p<0.001) were significant predictors for clinical pregnancy. Cut off value for the menstrual period was found to be 27.5 days with 82.6% sensitivity and 65% specificity. Cut off value for TETEMC was 11.5 days with 75% sensitivity and 63.2% specificity.

**Conclusion:**

Longer menstrual cycle and the TETEMC seem to be associated with the implantation failure.

## INTRODUCTION

Window of implantation is defined as the period of an optimal synchronization between the embryo and the endometrium. In physiological menstrual cycles, this period corresponds to the menstrual days of 21-24 days in a women with regular 28 days of cycles. In other words, implantation can be achieved in a period of 4-7 days to next expected cycle. This period of implantation is determined by the sensitive balanced stimulation of steroids hormones of estrogen and progesterone secreted through the cycle^(1,2,3)">(1,2,3)^. However; in stimulated cycles, it was reported that endometrial maturation can be 3 days early compared to unstimulated cycles^(4)^. In the current practice, window of implantation was tried to be predicted indirectly by endometrial thickness measurement. Cut off value for the endometrial thickness for successful implantation was reported to be 6 mm^(5,6,7)^. However according to our experience and the reports from literature, 50% of patients with optimal endometrial thickness and high grade embryos fail to conceive. Endometrial receptivity is determined by several factors and these factors were reported to be under the effect of gonadal hormones, so it is expected to see a change in receptive period with changing endocrine milieu. In ovarian stimulation cycles gonadal hormones are secreted in high levels compared to physiological states so this may change the implantation period.

The aim of this study was to assess whether the time from the menstrual day at embryo transfer to expected menstrual cycle (TETEMC) is associated with the implantation in women with regular cycles or not.

## MATERIALS AND METHODS

Between January 2014 and December 2015, women with regular cycles who underwent artificial reproduction in the in vitro fertilization (IVF)/intra-cytoplasmic sperm injection unit of Zeynep Kamil Women and Children’s Health Training and Research Hospital were recruited from prospectively collected database. Age, body mass index matched groups of women with (n=40) and without (n=40) successful implantation after grade 1 embryo transfer were randomly selected and compared in terms of some demographic and clinical characteristics including TETEMC, endometrial thickness at embryo transfer and duration of regular cycles. Embryo grading was determined according to the review by Alpha Scientists in Reproductive Medicine and European Society of Reproduction and Embryology Special Interest Group of Embryology^(8)^. All the participants had regular menstrual cycles, as well as normal serum prolactin levels and without hormone treatment within three months. The patients’ ages ranged from 24 to 39 years. In all patients artificial reproductive techniques (ART) were indicated for unexplained infertility. Unexplained infertility was diagnosed when a patient was infertile with normal ovulatory and tubal functions along with a normal sperm count for her partner. These were determined by the regularity of menstrual cycles, hysterosalphingography, and semen analysis, respectively. Women with low ovarian reserve, irregular cycles, polycystic ovarian syndrome and the endometriosis were excluded from the study.

Antagonist protocol was used in all cases; on the second day of the menstrual cycle, recombinant follicle stimulating hormone (rFSH), depending on patient’s response, were administered and follicular growth was monitored using transvaginal sonography. The dosage of rFSH was adjusted from day 5 of stimulation according to the ovarian response. Antagonist Cetrorelix (Merk-Sereno, Geneva, Switzerland) 0.25 mg/day was administered when the follicular size was 12 mm. After the follicular size reached >18 mm, recombinant human chorionic gonadotropin (HCG) 250 µg was administered, and follicular puncture was performed after 34-36 hours. Then we started the application of 8% vaginal progesterone gel twice/daily. Serum HCG level was measured two weeks later, and if serum HCG level was more than or equal to normal level, we performed ultrasonography to detect the pulse of fetus to confirm clinical pregnancy. TETEMC was divided into 4 groups as group 1: 0-4 days, group 2: 5-8 days, group 3: 9-13 days, group 4: >14 days. Groups were compared in terms of successful implantation.

TETEMC was the number of days from the day at embryo transfer to the first day of expected menstrual cycle determined from regular cycles.

### Statistical Analyses

Data was analyzed using SPSS 15.0 for Windows. Pearson’s correlation analysis or Spearman’s correlation analysis was performed to assess the correlation between different variables and ovarian response and the correlation between one variable and another as appropriate. Student t test was used to compare continuous variables between the groups. Multivariate regression analyses were used to assess the adjusted associations. Receiver operating characteristic (ROC) analyses were used to assess the predictive value of the test and to calculate sensitivity and specificity. P value <0.05 was accepted to be statistically significant.

## RESULTS

### Group comparisons

Comparison of groups with and without successful implantation revealed significant differences in between groups with regard to TETEMC and menstrual period (Table 1). There were 11 three day embryo transfers where as the number of five day embryo transfer was 69 (p>0.05).

### Correlation analyses

Correlation analyses revealed significant correlations in between the successful implantation and TETEMC, duration of menstruation and the age (Table 2).

### Multivariate regression analyses

Multivariate analysis revealed significant association in between the TETEMC and clinical pregnancy after adjustment for age and the duration of menstruation (Table 3).

### Receiver operating characteristic analyses

**Figure 1:**
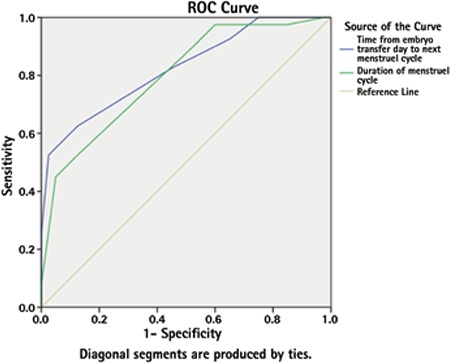
Receiver operating characteristic curve of transfer to expected menstrual cycle and menstrual period to predict implantation *ROC: Receiver operating characteristic*

In ROC analyses both the TETEMC (AUC=0.824, p<0.001) and the menstrual period (AUC=0.797, p<0.001, Figure 1) were significant predictors for clinical pregnancy. Cut off value for the menstrual cycle was found to be 27.5 days with 82.6% sensitivity and 65% specificity. Cut off value for TETEMC was 11.5 with 75% sensitivity and 63.2% specificity.

### Subgroup comparisons

Comparison of groups with TETEMC ≤11.5 and >11.5 days for successful implantation revealed a significant difference indicating higher rates in group with TETEMC ≤11.5 (75.9% vs. 35.3%, p<0.05, Table 4). Comparison of groups with duration from the menstrual period ≤27.5 and >28 days for successful implantation revealed a significant difference indicating higher rates in group with menstrual period ≤27.5 (82.6% vs. 36.8%, p<0.05, Table 5).

Comparison of successful implantation among the group with 4 different TETEMC revealed 100% implantation rate in group with TETEMC ≤8 days (Table 6).

## DISCUSSION

In this study, we tried to assess the effect of menstrual day at embryo transfer on the implantation rates in ovarian stimulation cycles. Our data revealed an early maturation of endometrium, however more sooner transfers especially 11.5 days before the next expected menstruation was associated with unsuccessful implantation with 75% sensitivity and 63.2% specificity. Besides estrogen and the progesterone, gonadotropin-releasing hormone (GnRH) receptors were shown in extra pituitary tissue including the endometrium^(9,10,11)^, studies reported the presence of GnRH mRNA gene expression in the endometrium throughout the menstrual cycle, with a significant increase in the secretory phase. These data indicate the possible physiological role of GnRH in the early stages of implantation via paracrine/autocrine pathways. Due to this physiological effect, clinicians have become suspicious for the possible negative effect of GnRH antagonists in combination with gonadotropin on the assisted reproductive technology success^(12,13)^. Some evidence showed detrimental effects of GnRH antagonist that may interfere with the embryo implantation. Consequently^(14)^, high dosages of GnRH antagonist (1 or 2 mg once daily) were found to be associated with low implantation rate (8.8 and 1.5%, respectively) in fresh cycles. Due to this data, in order to prevent ovarian hyper stimulation syndrome and for more receptive endometrium, freeze all policies were introduced and the review on this issue indicated reduced risk of ovarian hyper stimulation syndrome and improved outcomes with frozen embryo transfer^(15)^.

Reduced implantation rates in IVF cycles were shown in some studies compared to natural ones^(16)^, however, there is still some controversy regarding this issue. A large retrospective analysis, showed similar implantation rates between donor and recipient IVF patients^(17)^.

In IVF cycles, the day of oocyte retrieval was thought to be the equivalent to day 14 in a natural cycle in women with 28 days regular cycles^(18,19)^. However, in ovarian stimulation cycles, an advanced endometrial maturation has been shown in some studies, this advancement was reported to be around 2±4 days^(20)^ and seen in 45.5%^(21)^ of cycles. As a consequence, an early and increased progesterone concentrations were blamed for early secretory transformation^(22)^ and followed by mid-luteal glandular maturation arrest^(23)^. High serum estradiol concentrations in stimulated cycles were also thought to result in glandular ± stromal dyssynchrony that may interfere with the endometrial receptivity^(24)^. Another data showed the direct effect of HCG that might lead to the advanced endometrial maturation^(25,26)^. Finally, studies showed that ovarian stimulation changed the luteal phase endometrial development.

Luteal phase support was thought to significantly improve clinical outcomes in in-vitro fertilization cycles by the correction of these detrimental effects of ovulation induction^(27)^. There is a consensus on the detrimental effect of ovarian stimulation on the endometrial receptivity and some measures have been introduced to overcome this issue like luteal phase support however, we hypothesized that despite advanced endometrial maturation, earlier transfers may be the main problem that lead to failed implantation, therefore timing of embryo transfer may be the cornerstone of this problem.

Study assessed the histological features of endometrium both at the 6^th^ day after luteinising hormone (LH) surge and the 10 days after LH surge. Study revealed similar histological features with regard to endometrial maturation^(28)^, in another study, pinopodes were observed at 20th day of menstruation and indicated period of implantation window started to open at days of 22-23 in women with 28 day regular cycles^(29)^, as we mentioned above there is two to four days maturation advancement in stimulated cycles. Our data also showed some advancement in endometrial maturation but more sooner embryo transfers failed to implant. Significant predictive value of longer menstrual cycles also confirm this argument which increase possibility of high TETEMC.

A cochrane review on the comparison of ART success between the cases with two different embryo transfer days revealed significant difference in live birth rates in favour of blastocyst transfer (day 5 to 6) compared to cleavage stage transfer (day 2 to 3)^(30)^. This data supports our arguments that three days delay in timing of embryo transfer seem to increase success rate.

Recently published well designed study showed a suboptimal endometrial development in ART cycles, and indicated a altered regulation of specific endometrial receptors compared to the the natural cycle. Similar to our end point authors concluded to modify ovarian stimulation not only to yield the optimal number of oocytes, but also to achieve serum hormonal levels that promote an optimal endometrial development and better pregnancy outcomes with fresh cycles. In addition to this study proposed cancellation of fresh embryo transfer and vitrification of embryos and postponing the transfer to more suitable endometrial development such as reached during natural cycles or controlled endometrial maturation^(31)^. Our data showed that TETEMC lower than eight days resulted in 100% implantation where as there were 62.7% successful implantation in groups with TETEMC between the 9-13 days. The rate was 4.5% in group with TETEMC >13 days, we thought that this group of cases may be the appropriate candidates for freeze all policy.

Expression of HOXA10 varies in the human endometrium throughout the menstrual cycle, rising dramatically in the luteal phase at the time of implantation^(32)^. This pattern of expression suggests a role for HOXA10 in the process of cyclic endometrial development and endometrial receptivity.

We thought that there is a sensitive gene expression regulation during menstrual cycle that determines the duration of menstruation and the time of implantation window, with ovarian stimulation, it seems that this regulated gene expression is not easily adapt this new microenvironment, previous study indicated the minimum period required to achieve a new level is directly proportional to product half-lives because rates of decay control the ratio between the rate of synthesis and the concentration of gene products at steady state^(33)^.

Endometrial receptivity array have recently been introduced to assess the endometrial receptivity via genetic evaluation^(34)^, however this test needs invasive procedures.

## CONCLUSION

In conclusion, longer menstrual cycle and the TETEMC seem to be associated with the implantation failure. According to this data it is reasonable to take account the duration of regular menstruation and TETEMC to determine the candidates for freeze all policy.

## Figures and Tables

**Table 1:**
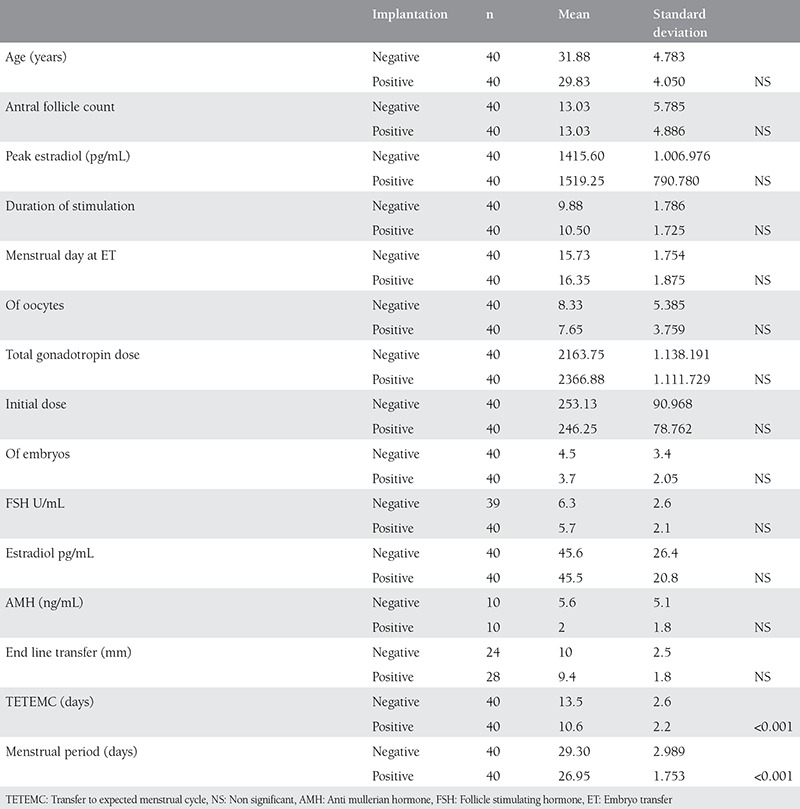
Comparison of some demographic and clinical characteristics between groups

**Table 2:**
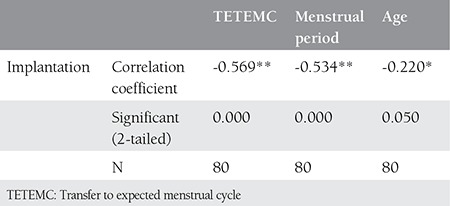
Summary of correlation analyses between successful implantation and some variables

**Table 3:**
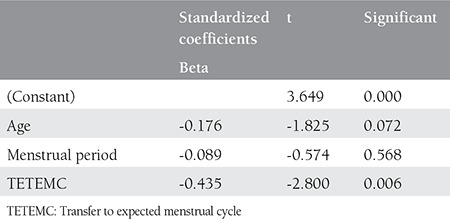
Multivariate regression analyses for successful implantation

**Table 4:**
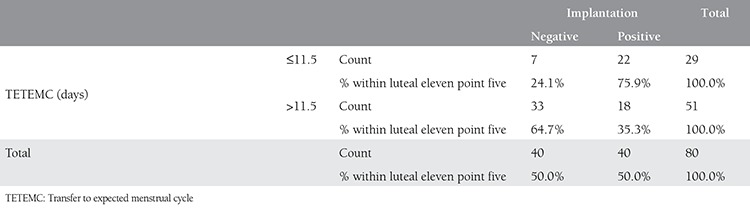
Comparison of implantation rates between groups with high and low transfer to expected menstrual cycle

**Table 5:**
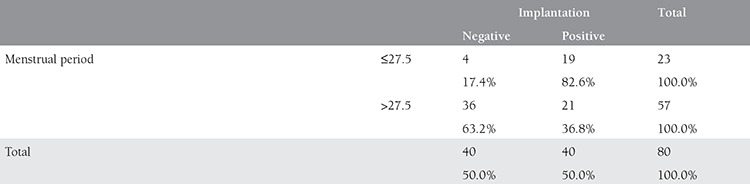
Comparison of implantation rates between groups with long and short menstrual period

**Table 6:**
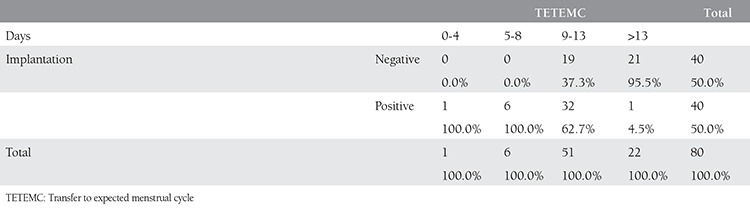
Comparison of implantation rates among groups with different transfer to expected menstrual cycle intervals
